# Nanomaterials and Annelid Immunity: A Comparative Survey to Reveal the Common Stress and Defense Responses of Two Sentinel Species to Nanomaterials in the Environment

**DOI:** 10.3390/biology9100307

**Published:** 2020-09-23

**Authors:** Kornélia Bodó, Nicoló Baranzini, Rossana Girardello, Bohdana Kokhanyuk, Péter Németh, Yuya Hayashi, Annalisa Grimaldi, Péter Engelmann

**Affiliations:** 1 Department of Immunology and Biotechnology, Clinical Center, Medical School, University of Pécs, Szigeti u, 12, 7643 Pécs, Hungary; bodo.kornelia@pte.hu (K.B.); kokhanyukb@gmail.com (B.K.); nemeth.peter@pte.hu (P.N.); 2 Department of Biotechnology and Life Science, University of Insubria, Via J.H. Dunant 3, 21100 Varese, Italy; n.baranzini@uninsubria.it (N.B.); rossanagirardello@yahoo.it (R.G.); 3 Quantitative Biology Unit, Luxembourg Institute of Health, 1A-B, rue Thomas Edison, L-1445 Strassen, Luxembourg; 4 Department of Molecular Biology and Genetics, Aarhus University, Gustav Wieds Vej 10, 8000 Aarhus C, Denmark; yuya.hayashi@mbg.au.dk

**Keywords:** nanoparticles, innate immunity, stress, earthworms, leeches

## Abstract

**Simple Summary:**

Nanotechnology is a dynamically developing field producing large amounts of nanocompounds that are applied in industry, daily life, and health care. During production, use, and waste these materials could end up in water or soil. Large scale contaminations of our environment are a threat to public health. Pollution can have harmful effects on the immune system, as revealed by numerous studies in humans and other vertebrates. The relative simplicity of invertebrate immune functions offers potentially sensitive and accessible means of monitoring the effects and complex interactions of nanoparticles which ultimately affect host resistance. Among terrestrial and freshwater invertebrates, earthworms and leeches are the “keystone” species to evaluate the health of our ecosystems. In this review we compare the conserved stress and immune responses of these invertebrate model organisms toward nanoparticles. The obtained knowledge provides exciting insights into the conserved molecular and cellular mechanisms of nanomaterial-related toxicity in invertebrates and vertebrates. Understanding the unique characteristics of engineered nanoproducts and their interactions with biological systems in our environment is essential to the safe realization of these materials in novel biomedical applications.

**Abstract:**

Earthworms and leeches are sentinel animals that represent the annelid phylum within terrestrial and freshwater ecosystems, respectively. One early stress signal in these organisms is related to innate immunity, but how nanomaterials affect it is poorly characterized. In this survey, we compare the latest literature on earthworm and leeches with examples of their molecular/cellular responses to inorganic (silver nanoparticles) and organic (carbon nanotubes) nanomaterials. A special focus is placed on the role of annelid immunocytes in the evolutionarily conserved antioxidant and immune mechanisms and protein corona formation and probable endocytosis pathways involved in nanomaterial uptake. Our summary helps to realize why these environmental sentinels are beneficial to study the potential detrimental effects of nanomaterials.

## 1. Introduction

Nanotechnology is an emerging field, which is claimed to be an ambassador in the new era of the industrial revolution [1,2,3]. Nanomaterials can have distinct physical properties and are also chemically more reactive than their bulk counterparts owing to their small sizes [4]. The number of engineered nanomaterials (ENMs) is steadily ascending; however, an additional increment is anticipated in the near future [2]. As a result of steady production and use, their deliberate or accidental discharge into the environment is of a particular concern for the health of the ecosystem and the human population [5,6]. In the environment, the toxicity of ENMs mainly depends upon their size distribution, chemical composition, surface charge, coating, shape, possible contaminants, and other physical properties [7].

## 2. Risk Assessment of Silver Nanoparticles to Soil Invertebrates

Silver nanoparticles (AgNPs) in the size range of <100 nm are the most abundant metal-based ENMs and are increasingly being employed in industries, households, and commercial applications [8,9,10]. Despite their conceded antimicrobial attributes, several previous studies have reported on AgNPs’ “eco”-toxicity, which is frequently connected to the dissolved Ag ions (Ag^+^) released from AgNPs during the surface oxidation [7,11,12,13,14,15]. Nevertheless, it is also accepted that physico-chemical features of AgNPs have a substantial influence on ecotoxicity. For instance, previously it was confirmed that uncoated AgNPs are more toxic to soil-dwelling animals than their coated counterparts [16,17]. With the increment in Ag-based goods in our environment and daily life, it is fundamental to inquire about the possible toxicity of AgNPs and Ag^+^ towards living organisms under multiple circumstances.

So far, bacteria, aquatic invertebrates, and soil-dwelling invertebrates have played prominent roles in nanotoxicology studies, since these organisms are among the first to encounter nanoparticles in our environment [4,18]. Several in vitro and in vivo studies (applying diverse cell lines and organisms) have dealt with the toxicity of AgNPs; however, the effects of particles on even closely-related species (in the sense of biochemical and immunological processes) are often debatable and ambiguous [10,19,20,21]. Furthermore, the use of AgNPs in different sizes and coatings makes these studies more complicated to compare. Among soil invertebrates, earthworms as key sentinel species are widely applied in soil nanotoxicity. They are often chronically exposed to contaminants via the epidermis and the gastrointestinal tract because of the ingested soil [22,23]. Earthworms possess relatively high heavy metal resistance and are also able to temporarily store and inactivate heavy metal ions; therefore, they are intensely impacted by soil contaminants [24,25]. Their responses towards metal exposures are evincible at several biological levels, from genetic to cellular [26,27]. During phylogenesis, differentiated mesodermal tissues and body (coelomic) cavities developed initially in these animals, and this progression makes earthworms more suitable toxicological models for cross-species comparisons with higher developed animals or vertebrates [12,28].

To date, most results have emphasized the endpoints of AgNPs’ toxicity in earthworms to individuals’ physiological parameters for comprehending the mortality following chronic or acute exposures, cocoon production and reproduction, bioaccumulation, and occasionally growth or behavior [17,23,29,30,31]. During these in vivo examinations in diverse type of soils, ionic Ag (Ag^+^ from AgNO_3_, used as a positive reference) was more toxic to earthworms than the NP counterpart. AgNPs also have noxious impacts on animals at higher concentrations [32,33]. Regarding in vivo studies we must consider the complexity of soil components and soil matrix properties with the soil type (e.g., organic matter, cations, moisture, water retention capacity, ionic strength, and fluctuating pH), because these factors may influence the behavior of NPs in soil (e.g., higher propensity to heteroagglomeration/homoagglomeration, change in morphology, and dissolution) [14,34].

### 2.1. AgNP Toxicity at the Organismal Level

In the course of in vivo earthworm studies assessing different endpoints (e.g., survival, reproduction, bioaccumulation, apoptosis, and mortality) AgNPs exerted harmful effects at the individual level. The majority of the current studies (Table 1) have applied *Eisenia fetida* (*Oligochaeta*, *Lumbricidae*) as a model organism [35,36] to monitor the AgNPs’ deleterious consequences; however, several studies also exist that applied other earthworm species [14,17,23,29,30,31,32,33,37,38]. The same amount of coated AgNPs (with either polyvinylpyrrolidone (PVP) or oleic acids) or ionic silver caused no remarkable impacts on mortality and growth in *E. fetida* exposed in soil. The reproduction was significantly reduced in earthworms exposed to ionic silver (94.21 mg/kg) and to higher concentrations of AgNP (773.3 mg/kg PVP-coated and 727.6 mg/kg for oleic acid-coated AgNPs). During the course of AgNPs’ accumulation and toxicity tests, the authors did not observe any coating-related differences in earthworms’ parameters [11]. It was also shown that after 48 h, *E. fetida* consequently avoided soils containing AgNP (10 and 30–50 nm with PVP-coating) and the equivalent concentration of Ag^+^ [16]. Gomes et al. [32] observed 100% survival of tested *Enchytraeus albidus* worms upon exposure to 30–50 nm PVP-coated AgNP (up to 1000 mg/kg soil), but the exposures resulted in a 50% reduction of juveniles at approximately 225 mg/kg. Brami et al. [31] suggested that *Allolobophora chlorotica* earthworms are more susceptible to AgNPs compared to *E. andrei* and *E. fetida*. A concentration-dependent mortality (below 40%) of *A. chlorotica* was recorded at lower AgNP concentrations (e.g., 50 and 125 mg/kg soil) after 14 d; however, complete death of the tested earthworms was found in the range of 250–1000 mg/kg soil concentration. Furthermore, even at the lowest applied concentrations (12.5 mg/kg), elevated avoidance behavior was detected. The presumed sensitivity among the closely-related species is likely to be in connection with the higher Ag accumulation in earthworms; as a consequence, increased mortality can also be seen [16]. Based on these observations, *Eisenia* spp. are not always the most appropriate model under standard laboratory conditions, because they might be less sensitive than other members of the *Lumbricidae* (*Annelida*, *Oligochaeta*) family. This phenomenon is presumably the outcome of their natural needs or habitat formed by evolutionary processes [27,39]. Exposure of *Lumbricus terrestris* earthworms to 20 nm (±2.5 nm) AgNPs led to increased mortality and caused elevated apoptotic responses in the exposed tissues (cuticle, intestinal epithelium, and chloragogenous tissue) [23]. In *Enchytraeus crypticus* the effect of 15 nm Ag NM-300K and Ag^+^ was thoroughly examined by Comet assays, and higher dose-dependent DNA damage was shown following three days of exposure to Ag^+^. Contrastingly, the Ag NM-300K resulted in more pronounced genotoxicity even at the lowest concentration (60 mg/kg soil) after 7 d, indicating non-monotonic dose-response toxicity [40]. Using *L. rubellus* earthworms, no apparent perceivable differences in activity, locomotion, or survival were observed upon 50 nm AgNP (500 mg/kg soil) treatment in the course of the 96 h exposure time (Table 1). Despite their survival, AgNPs were detected in setae, follicles, nephridia, and chloragogenous tissue after 168 h [30]. These results shed more light on the relevance of the AgNPs’ characteristics/physico-chemical qualities regarding their toxicity to earthworms at the organismal level.

### 2.2. AgNP Toxicity at the Sub-Organismal Level (Stress and Defense Mechanisms)

There are only a limited number of AgNP-related studies focusing on cellular/sub-cellular molecular endpoints in earthworms (Table 1) [12,27,40,41,42,43,44,45,46,47,48]. At the cellular or sub-cellular level, most of the aforementioned studies are related to the free circulating coelomocytes that are considered macrophage-like cells contributing to the “cellular arm” of earthworm innate immunity [49,50].

Recently, it has also been clarified that coelomocytes’ subpopulations (amoebocytes and eleocytes) can be distinguished based on their diverse morphologies and functions such as phagocytosis and the production of bioactive molecules [50]. We previously examined the interactions of amoebocytes and eleocytes after 24 h of incubation with FITC-conjugated *Escherichia coli* and *Staphylococcus aureus* bacteria in vitro, where we found that amoebocytes were able to engulf significantly more bacteria than eleocytes [49]. Amoebocytes as effector immune cells also express evolutionarily-conserved pattern recognition receptors (PRRs) that detect pathogen-associated molecular patterns (PAMPs) [51]. Gene expression patterns of PRRs are irrefutably different between amoebocytes and eleocytes. Several *PRR*s, immunologically and oxidative stress-related genes (*CCF*, *TLR*, *lumbricin*, *LuRP*, *MyD88*, *copper/zinc and manganese SOD*) were expressed only in the amoebocyte subpopulation. In contrast, other genes such as antimicrobial or bioactive molecules (*lysenin*) and lysosomal hydrolases (*cathepsin L* and *cathepsin B*) were induced in both subpopulations but were at relatively higher levels in eleocytes [52]. Eleocytes contribute to the humoral immunological processes mainly through their lysenin secretion and they possess an indispensable role in the homeostasis of the host earthworms [49,50].

Due to the phylogenetically-conserved role as phagocytes to combat exogenous entities, those immune cells are of particular interest to studying NP-cell interactions [12,50]. In this context, earthworm coelomocytes are applicable as a simple, invertebrate in vitro model to evaluate the toxicological or immune-toxicological end-points at the cellular and molecular levels upon exposure to NPs and other environmental contaminants [12,27,41,44,48]. That said, only a little is known about the potential impacts of AgNPs on the immune systems of soil invertebrates in general. In recent years, a number of studies observed the complex temporal stress responses at the cellular and molecular levels to AgNPs [12,27,41,44,45]. Several in vitro models of higher vertebrates reported that stress responses gave rise to disturbance of redox equilibrium resulting in inflammation, genotoxicity, and cytotoxicity [53,54,55].

**Figure 1:**
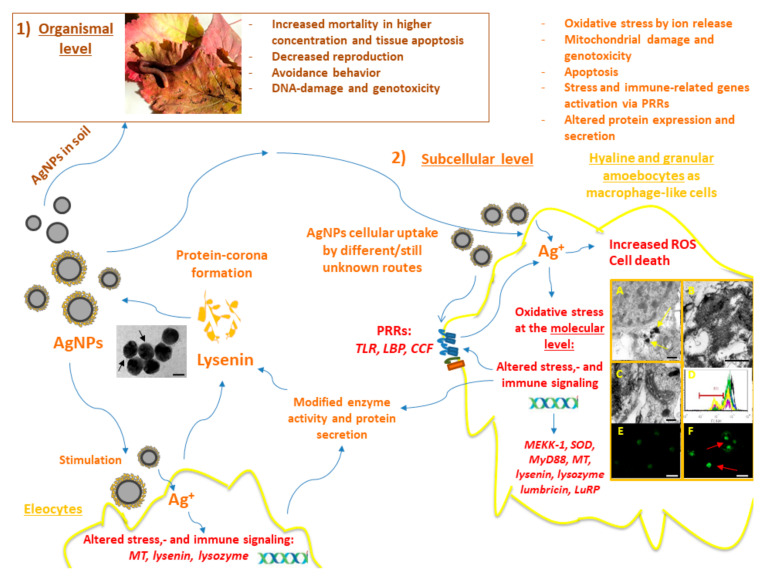
Schematic overview of the AgNPs’ impacts on earthworms at different biological levels. (**1**) AgNP toxicity at the organismal level: The representative image depicts an *E. fetida* earthworm. Adjacent to the image, the deleterious effects of AgNPs known from the in vivo earthworm experimental literature are listed. (**2**) AgNP toxicity at the sub-cellular level: Different endpoints of AgNP toxicity are detailed in the upper right corner. Blue arrows demonstrate the directionality and impacts of AgNPs. AgNPs can interact with both amoebocytes and eleocytes. Recognition of AgNPs in target amoebocytes is achieved probably by an assortment of PRRs, and various endocytosis pathways are engaged (please see the main text and Figure 2). Our studies revealed that lysenins are the key constituents of AgNP-protein corona from earthworm coelomocytes. The representative TEM image shows the coelomic fluid-derived protein coating of AgNPs (black arrows, scale bar: 50 nm). AgNPs caused irreversible alterations in amoebocytes, in vitro; in the cytoplasm, AgNP aggregates (**A**) (yellow arrows) can be observed by TEM (scale bar: 200 nm). Apoptotic nucleus (**B**) and atypical mitochondria (**C**) can be seen in amoebocytes exposed to AgNPs revealed by TEM (scale bars: 500 nm, 200 nm). Mitochondrial membrane potential was significantly decreased (**D**), as indicated by MitoView fluorescent dye-based flow cytometry (representative histogram) (black line—control, red line—15 µg/mL AgNP, blue line—30 µg/mL AgNP, purple line—40 µg/mL µg/mL AgNP, green line—20 µg/mL AuNP, yellow line—1.35 µg/mL AgNO_3_). TUNEL assay demonstrated TUNEL^−^ (**E**) and TUNEL^+^ (**F**) apoptotic nuclei from control or AgNP exposed coelomocytes, respectively (red arrows, scale bars: 20 µm). In Vitro, the lower-cytotoxic concentrations of AgNPs at the molecular level can manifest in biased gene expression of stress-related factors and immunologically-active secretory proteins (such as lysenins). These gene expression profiles signify the involvement of antioxidant systems such as *SOD*, and persistent up-regulation of *MT* in *Eisenia* underlining the thiol-mediated detoxification process. The prompt regulation of *TLR* to AgNPs was apparent in *E. fetida* coelomocytes as an NP-specific response.

Initially, Hayashi et al. [12] investigated the conserved biological processes comparing *E. fetida* earthworm coelomocytes and THP-1 cells (a human acute monocytic leukemia cell line) exposed to AgNP (PVP-coated, 83 ± 22 nm, ranged 0–5.91 μg Ag/mL) both at the cellular and at the molecular level, in vitro. Interestingly, in this study, the cell viability of earthworm coelomocytes followed a similar concentration–response curve as that of differentiated/macrophage-like THP-1 cells, rather than that of undifferentiated/monocytic THP-1 cells. This in vitro study also confirmed by electron microscopy the intracellular accumulation of AgNPs—and related toxicity—in the phagocytic coelomocytes (Figure 1A–C) [12]. Subsequently, temporal aspects of oxidative stress and immune-related biomarker gene expression were investigated. The obtained results proposed the early control of oxidative stress response (*MEKK-1*, *HSP70*, and *Cat*) genes and the later induction of immune signaling associated genes, such as *MyD88* in earthworm coelomocytes and THP-1 cells [12]. These in vitro results further strengthen the connection between the highly conserved stress response and innate immune signaling mechanisms across the animal kingdom.

In the in vivo experimental counterpart, the main focus was on the overall time-course profiling of stress and immune-related genes and enzyme activities as biomarkers for oxidative and metabolic responses. Intriguingly, the gene response patterns resulting from AgNP and Ag^+^ treatments were different over time. Ag^+^ conspicuously triggered a faster response (1 d of exposure) in genes (such as *SOD*) and enzyme activity related to ROS catabolism. Overall, however, both Ag forms exerted an influence on stress responses (AgNPs only after 2 and 7 d affected the *Cat*, *SOD*, *HSP70*, and *MEKK1* genes) [41]. Similar temporal aspects in the expression of oxidative stress-related genes were recorded in *E. fetida* exposed to AgNPs. This study emphasized that the distinct mechanism of AgNP and Ag^+^ toxicity may be connected to the kinetics of exposures [43]. Earlier molecular aspects of Ag^+^ and AgNP exposures in *E. fetida* associated initially with oxidative stress response and metabolism, while slower effects were observed for innate immunity (such as *MyD88* and *lysozyme*) [12,27,41]. In another in vitro study, the association between oxidative stress and immune activation was not entirely apparent using *E. fetida* coelomocytes exposed to mildly-cytotoxic Ag NM-300K and Ag^+^ concentrations (EC_10_). Nonetheless, the *TLR*-related differential gene expression (concurrent induction with *LBP/BPI*) at 24 h was clearly different for NM-300K and Ag^+^ exposures, which suggests NP–coelomocyte interactions [46].

Gomes et al. [42] investigated stress biomarkers using *E. fetida* following in vivo Ag^+^ and AgNP exposure conditions and observed similar facts in stress response, with some specific time-related differences. Following 4 d of exposure to AgNPs the increased total glutathione (TG) denoted that the earthworms were responding to oxidative stress. The early induction of glutathione-S-transferase (GST) and glutathione peroxidase (GPx) supported the privileged role of the antioxidant system. Afterwards, the eventuating inhibition might have been due to the high affinity of Ag^+^ for thiol groups of cysteine-rich proteins and enzymes, because the cellular thiol-depletion results in the inactivation or collapse of the antioxidant defense processes [56]. Despite the effects on antioxidant system that were common to both Ag forms, the increment activity in lipid peroxidation following 28 d of treatment could also be seen, underlying the gradual manifestation of oxidative impairment [42]. In nanosilver pathophysiology, these outcomes highlight the cross-talk from oxidative stress-associated metabolisms regarding the activation of the immune response with the apparent involvement of immune-competent cells based on in vitro and in vivo approaches.

### 2.3. Species-Specific Differences in AgNP Toxicity

Even though several studies dealt with Ag^+^ or AgNPs toxicity, not much is known about the differences in sensitivity of closely-related species to those similar harmful materials. Therefore, relying on our previous AgNP-based data [12,41,45,46], we have compared the immune and toxicity mechanisms of two noble metal NPs (10 nm-sized PVP-coated AgNPs and AuNPs, and Ag^+^ as reference) in the coelomocytes of two closely-related *Eisenia* species, in vitro. Since *E. andrei* and *E. fetida* earthworms have widely been used as models in ecotoxicology studies and as we are aware that their natural habitat is fundamentally different [39], we hypothesized that species-specific variations may exist beyond the cellular and molecular mechanisms [27]. As discussed earlier, oxidative stress was found to be involved in AgNP toxicity, which we investigated in our study with concentrations of AgNPs and AuNPs of up to 40 µg/mL. At the sub-cellular level, the cellular redox reactions upon exposure to AgNPs increased in a dose and time-resolving manner, leading to specific impacts, including mitochondrial damage (Figure 1D) and caspase-3 activation in connection with apoptosis and the genotoxic response (Figure 1E,F) in the coelomocytes of both closely-related earthworm species (Figure 1). Similar effects were not obtained following the AuNP treatments. Furthermore, *E. fetida* coelomocytes demonstrated greater sensitivity and apoptosis-responses compared to *E. andrei* coelomocytes towards AgNPs [27]. Coinciding with the previous approach [41,45,46], mildly-cytotoxic concentrations of AgNP and Ag^+^ (EC_20_ values) were chosen for the assessments of species-specific responses, including temporal aspects of gene expression patterns, ex situ protein corona formation, and protein secretion (mainly focusing on lysenin). Temporal profiling of transcriptional responses unveiled clear differences over time (2, 12, and 24 h) between *E. andrei* and *E. fetida* coelomocytes, supporting the greater susceptibility of *E. fetida* coelomocytes to AgNPs. Overall in *E. fetida*, the most distinguished effect of AgNPs was observed in the early induction (2 h) of *TLR* that provoked continuously increased expression in *SOD* and *MT* for redox and metal regulation towards 24 h [27]. In addition, a progressive and contrasting regulation in the *lysenin* expression over time was common to both species. In contrast to studies related to AuNP toxicity [20,57], no cytotoxicity in *Eisenia* coelomocytes could be detected, but it modified the expression-pattern of immune-response genes (e.g., *TLR*, *lysenin*) [27].

In *Eisenia* earthworms, lysenin is a multifunctional key protein element of coelomic-fluid (widely employed as biofluid) produced by eleocytes; however, its appearance in the *Lumbricidae* family is not consistent because it was not identified in *Dendrobaena venata (E. hortensis)*, *L. terrestris*, *L. rubellus*, or *Aporrectodea caliginosa* [58]. Using *Eisenia* coelomocytes in vitro, AgNPs and AuNPs suppressed expression of *lysenin* over time and the observations were also supported by results at the protein level. For both species, lysenin at the protein level accompanied the differentially expressed *lysenin* gene; therefore, by following 4 h exposure to AgNPs and AuNPs, we observed elevated lysenin secretion, but when nearing 24 h a decreased pattern could be seen compared to untreated cells [27]. AuNPs-related effects are still less known compared to those of AgNPs; therefore, that requires further research, wherein the main focus will be on mildly-cytotoxic concentrations and the plausible altered immunity following changes at the protein level.

### 2.4. AgNP-Associated Biomolecular Corona Formation in Earthworms

Nanomaterials can enter the host organisms by different routes (e.g., ingestion, inhalation, or intracutaneous). Regardless of the way of body entry or in cell culture media, NPs bind diverse biomolecules that result in the formation of bio-molecular coronas that may have impacts on their behavior and how they interact with cells. The parameters influencing the protein corona (PC) composition are particle size, shape, charge, and other surface properties, and the composition of biological fluid and its qualities [59]. The newly formed PC provides NPs a biological identity; they can present a discernible molecular pattern that determines the bioavailability, colloidal stability, biodistribution, cellular uptake, toxicity, and clearance from the body. What makes it more complicated is the process of dynamic PC formation, as it also depends on the competition between proteins adsorbed on the surfaces of NPs [60,61,62].

The study on NP-protein coronas in invertebrates is hampered by the lack of knowledge on the exact compositions of biological fluids (e.g., coelomic fluid, hemolymph), because of the limited available information thanks to the vast number of invertebrate species. The contrast in the compositions between mammalian blood and invertebrate blood is quite stark because mammalian blood comprises large numbers of well-studied proteins (albumin, immunoglobulins, complement proteins, etc.) that participate in the formation of a bio-molecular corona around NPs [59].

It is thus expected that the formation of NP-protein coronas in invertebrates varies significantly across different species simply due to the inherent difference in protein repertoires within the bodily fluids [60]. In mammalian blood, and by saying this we refer to the aforementioned common proteins such as albumins, the PC formation is often considered as a generic guarding force against the toxicity of NPs, because the binding of proteins to NPs effectively reduces the surface energy and thus prevents non-specific physical interactions with, e.g., cell membranes [63]. Generally, the extracellular fluid of invertebrate species has significantly lower protein content because of their immunity comprising only the innate immune system (i.e., the lack of immunoglobulins) [64]. There are nonetheless studies emerging for soil-dwelling (earthworms) and marine (mussels) animals, which pointing out that the knowledge from mammalian experiments cannot be directly applied to invertebrates because the NP-protein coronas in those organisms are rather species-specific and can be dominated by a number of unique proteins with high affinities for NPs [46,65,66].

The first presentation of AgNP-related protein coronas in invertebrates was derived from the bodily fluid of *E. fetida* earthworms and the cell culture supernatant of their coelomocytes [45]. Furthermore, it became clear that lysenin, a major protein component of the coelomic fluid with known immune functions, was strongly bound to AgNPs (including NM-300K and a larger, 75 nm AgNP), facilitating the protein corona-directed accumulation in the coelomocytes [46]. Due to the observed differences at the gene level in our recent work [27], we further explored the ex situ binding selectivity of *E. andrei* and *E. fetida* coelomic proteins. Besides AgNPs, we used AuNPs for comparison as they possess similar chemical attributes to AgNPs in terms of the surface reactivity with thiols. Despite the high BSA background, the specific enrichment of lysenin proteins in PC could be observed only in the case of AgNPs; therefore, similar substitution is likely to appear in the presence of cell cultures and under exposure conditions [27]. In addition, the study has given a preliminary confirmation that different compositions in the PC can be found even between closely-related species due to the inherent species-specific heterogeneity in the protein repertoire, whereby in lysenin-related protein 2 is specific only in *E. fetida*. In both species, expression/secretion of lysenins appears to be stress-regulated and this refers to a multiple feedback mechanism for AgNPs because lysenins are particularly enriched by AgNPs and known to enhance uptake by earthworm coelomocytes [46]. In light of those facts, knowledge on the molecular interactions happening at the NP-bio-interface embodies an opportunity to comprehend the basis for the biological activities of these complexes in both invertebrate and vertebrate organisms.

## 3. Routes of AgNP Uptake in Invertebrates

The interaction of NPs and immune cells, be it through the protein corona or not, in general leads to uptake and/or activation of signaling cascades downstream of receptor ligation. Here we focus on the routes of NP uptake in invertebrates, for which some insights are available in the literature that may also be relevant in understanding the very process in annelids. According to the general classification for eukaryotes, there are two major endocytic pathways, such as phagocytosis and pinocytosis that can be distinguished based on the cell type-specificity and target entities that are taken up [67].

Phagocytosis is involved in the immune function against large non-self elements of biological (bacteria and viruses) and chemical (including microparticles) origins and is performed mostly by professional immunocytes, such as macrophages, monocytes, dendritic cells, and neutrophils [68]. Classically, the process starts with the recognition of opsonized particles, and proceeds to actin-driven ingestion and the forming of the phagolysosome [67].

Macropinocytosis is another type of actin-dependent bulk engulfment, but it differs from phagocytosis in that it captures solutes in a non-specific manner. During macropinocytosis, membrane protrusions with the help of actin fuse around the solutes, forming 1 µm or bigger macropinosomes, which then merge with lysosomes.

**Figure 2:**
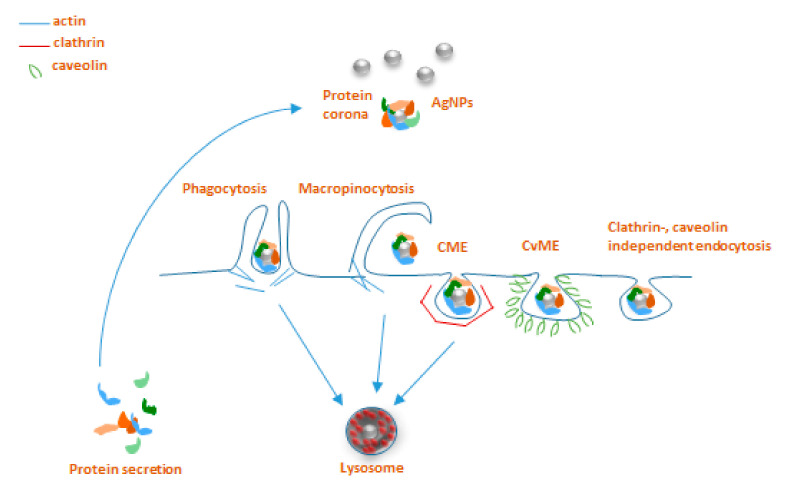
Main endocytosis pathways possibly involved in internalization of AgNPs: phagocytosis, macropinocytosis, clathrin-mediated endocytosis (CME), caveolin-mediated endocytosis (CvME), and clathrin/caveolin-independent endocytosis.

Practically, all types of cells are capable of endocytosis, which takes place by at least four different pathways, including micropinocytosis, clathrin-mediated endocytosis (CME), caveolin-mediated endocytosis (CvME), and clathrin/caveolin-independent endocytosis [67,69]. Briefly, internalization by CME occurs with the help of clathrin, which forms a basket-like structure at the cell membrane, typically around 150 nm in size [67]. This process, just like phagocytosis, leads to the fusion with lysosomes forming endolysosomes [69,70]. CvME is characterized by the formation of 50–80 nm flask-shaped membrane invaginations, the main component of which is caveolin, a cholesterol-binding protein. In contrast to abovementioned pathways, a formed caveosome does not contain enzymatic proteins, and the vesicle does not go through lysosomal degradation [67,70,71]. Among the clathrin, caveolin-independent endocytic pathways, particularly well described is a mechanism involving 40–50 nm sized cholesterol-rich lipid “rafts”, which participate in sorting of intracellular molecules [72]; however, these pathways are not reported to be extensively involved in NP uptake (Figure 2) [73].

Very limited data are available about the involvement of particular endocytosis pathways for AgNP uptake in invertebrates, with practically no information on these mechanisms for earthworms. On the other hand, somewhat better unraveled AgNPs endocytosis mechanisms have been reported for other invertebrate species of nematodes, snails, and mussels. These studies applied various in vivo and in vitro approaches using pharmacological inhibitors originally developed for use in vertebrates.

Endocytic mechanisms of the nematode *Caenorhabditis elegans*, one of the classical animal research models, are among the most deeply studied, compared to other invertebrates. Maurer et al. [74] investigated the uptake pathways for citrate-coated 25 nm AgNPs using both pharmacological inhibitors and endocytosis-deficient mutants. Interestingly, the CME inhibitor chlorpromazine almost entirely abolished the AgNP-related toxicity, indicating the importance of CME in AgNPs uptake in nematodes. Moreover, the research also provided evidence for decreased sensitivity of endocytosis-deficient mutants that accumulated fewer AgNPs than did the wild-type nematodes.

Khan and co-workers [75] revealed, by exposing of estuarine snail *Peringia ulvae* to 18 nm AgNPs in aqueous solution, that AgNPs uptake was largely affected by inhibition of CME by amantadine. Blocking CvME and the channel-coupled H^+^ pump also reduced the internalization, but only after 24 h of exposure, indicating the possible differences in the uptake kinetics via different pathways. Phenylarsine oxide (non-specific inhibitor of macropinocytosis) affected the AgNPs uptake after 6 h exposure, which might result from activation of macropinocytosis as a compensatory pathway when the other mechanisms are less active.

Recent research on *Mytilus galloprovincialis* mussel hemocytes discusses the connection between different endocytic pathways, AgNP cytotoxicity, and immunocyte counts [76,77]. Upon the blocking of CME by amantadine, small AgNPs (<50 nm) caused slight toxicity to hemocytes. A similar tendency was observed for AgNPs of a bigger size (>100 nm); when CvME was inhibited by nystatin, AgNPs caused only minor toxicity to those cells. This is controversial, considering the general opinion that 40–50 nm NPs are internalized by CvME, while bigger (100–120 nm) NPs are taken up by CME. Nevertheless, the endocytosis inhibitors changed the sensitivity of hemocytes towards the AgNPs, mainly showing a delayed effect in the onset of NP toxicity. Notably, none of them completely blocked AgNP uptake and related cytotoxic effects, suggesting that several uptake pathways participate in the process parallelly or by compensating for each other. Besides, in these studies, the AgNP content was measured in whole animal tissues and thus did not elucidate the role of invertebrate immunocytes in AgNP uptake, which are the first responders to foreign particles that infiltrate the body of the host organism.

One of the limited studies on this topic investigated the transcriptomic changes of AgNP exposure in the earthworm *E. fetida* [78]. It brought interesting results of upregulation of genes coding for endocytosis and functions of cell cilia. Earlier there was evidence that so-called “ciliary pockets” in the invertebrates’ epithelial membranes are involved in CME [79], which might indicate the involvement of this mechanism in earthworms as well.

To conclude, the summarized studies give an understanding of evolutionarily conserved processes potentially participating in AgNP uptake in annelids. Nevertheless, the area needs further investigations to obtain the complete picture of the NP entry to the cell and the immune responses in invertebrates.

## 4. Carbon Nanotubes and Related Toxicological Risks

In the last few decades, the development of nanotechnologies led to the discovery and production of several new nanomaterials that have recently acquired numerous roles, especially in industry. Among them, carbon nanotubes (CNTs), which consist of concentric graphene tubes with diameters of 0.7 to 100 nm, are classified as single-walled (SWCNTs) and multiwalled (MWCNTs) carbon nanotubes and represent one of the most promising groups for industrial development, nanoelectronics, mechanical engineering, and biomedical applications [80,81]. Moreover, as their functions are constantly improving, these nanoparticles are also gaining importance in the biological field. Indeed, although their discovery produced both social and economic benefits, their manufacturing corresponds to a rapid expansion of their environmental discharge. In particular, due to their non-biodegradable characteristics, their presence has been observed both in air and in soil, and often CNTs are also released into the surface water (such as rivers and lakes), posing potential risks to human and animal health.

A number of studies recently demonstrated both the toxicity and the bio-persistence of CNTs within tissues and cells [82,83,84], and these elements are connected with their morphology, which is a crucial aspect for the development of human diseases, such as mesothelial injuries and carcinogenesis [85]. These features are very similar to those of asbestos fibers, showing asbestos-like pathogenicity [86,87]. In fact, it has been observed that CNTs also induce frustrated phagocytosis [88] and cause the release of pro-inflammatory cytokines and reactive oxygen species (ROS) [89]. They possess a very long half-life in vivo and can affect the cellular functions after physically penetrating the biological barriers and causing chronic inflammation, which is often connected with cancer insurgence [5]. Interestingly, all these data point out that the immune system and ROS production represent sensitive physiological indicators following exposure to CNTs, even at low concentrations.

In this context, the necessity to develop and optimize new approaches for investigating the effects of CNTs suggests the use of specific research models and the choice falls back on those normally employed in ecotoxicological studies.

### 4.1. Carbon Nanotubes and Aquatic Invertebrates

Invertebrates attracted great interest thanks to the possibility of obtaining rapid and secure evaluations. The use of some species rapidly increased in the last decades, replacing vertebrates in many research fields. The benefits are related both to their anatomical and to their physiological characteristics that allow one to easily analyze many biological processes, and to their low complexity at the genetic, cellular, and molecular levels. In particular, the toxic effects of CNTs released in the ecosystems [90], whose predicted environmental concentrations (PECs) in aqueous systems are projected to approximately be 0.001–1000 μg/L [91], have been mostly investigated in aquatic invertebrates, especially in those from marine environments. It is well-known that these animals coordinate fast and sensitive reactions, especially those associated with acute behavioral or developmental responses, enzymatic activity alterations, regenerative capacity, respiration rate, and biochemical performance, following exposure to CNTs. Indeed, exposure to carbon-based nanomaterials causes in *Artemia salina* inhibition of larval swimming and alterations in the enzyme activities in a concentration–dependent manner [92]. Moreover, MWCNTs induced negative effects on the regenerative capacity in the polychaete *Diopatra neapolitana*. Additionally, higher MWCNT concentrations induce energy-related responses characterized by higher values of electron transport system activity, glycogen, and protein concentrations in both the polychaete species *D. neapolitana* and *Hediste diversicolor* exposed to this contaminant. Furthermore, oxidative stress with higher lipid peroxidation, lower ratios between reduced and oxidized glutathione, and higher activity of antioxidant and biotransformation enzymes are detectable in those organisms exposed to MWCNTs [93]. A MWCNT concentration-dependent effect is also evident in larval development in the two different populations (Mediterranean and Atlantic) of polychaete *Ficopomatus enigmaticus* [94]. Surprisingly, an unexpected decrease in the effect, in terms of interference with the correct development of larvae, was observed at the highest exposure concentration of MWCNTs (9.00 mg/L). This apparent reduction in the toxic effect at high concentrations is probably due to the formation of large MWCNT aggregates that, through precipitating on the seabed by gravitational sedimentation [95,96], interact less with the larvae.

Sedimented MWCNT aggregates are, however, a cause of several toxic effects on benthic animals living on or in the seabed, such as adult polychaetes and mollusk bivalves. Indeed, studies performed on *H. diversicolor* clearly demonstrate that the accumulation of MWCNT aggregates provokes neurotoxicity, alters energy-related biochemical processes, and activates antioxidant defenses and biotransformation mechanisms [93]. Moreover, the MWCNT aggregates sedimented by the water column have their capture by the gills of bivalves facilitated [97]. Indeed, due to the feeding by filtering, nanoparticles are trapped by gills of bivalves, flow into the gut and the digestive gland, and are translocated from the gut into the hemolymph accumulating inside the body. Large MWCNT aggregates, observed in the intestinal lumen, in the tubules of the digestive gland and gills of several species of bivalves (*Crenomytilus grayanus*, *Swiftopecten swifti*, *Modiolus modiolus*), induce significant morphological organ damages, such as erosion and necrosis of the epithelium, increased vacuolization and apoptosis of the cells, and swelling of the connective tissue [98,99]. The contact of nanomaterials with the target tissues not only involves physical damages but also provokes oxidative stress in the cells, by directly inducing the production of ROS [100]. Moreover, evaluation of the hemolymph and circulating hemocytes reveals that MWCNTs may cause immunotoxicity in some bivalve species. Indeed, after 48 h exposure to MWCNTs, the total hemocyte count in the scallops *S. swifti* was significantly reduced while the average hemocyte granularity greatly increased. These effects could be caused not only by the death of hemocytes due to a negative influence of NPs but may also be related to mass migration of hemocytes from circulation towards other tissues for elimination of NPs. The increased hemocyte granularity is, on the other hand, probably connected with phagocytic clearance of NPs in the affected organs [98].

### 4.2. Carbon Nanomaterials and Leeches

Among aquatic invertebrates, the medicinal leech (*Hirudinea*), besides representing a suitable model that avoids the vertebrate ethical restrictions, is considered as an ideal environmental biomarker that permits one to develop easy and rapid methods for evaluating effects of the dispersed MWCNTs in aquatic environments [101,102]. It is well-known that these animals coordinate the immune system and the inflammatory response upon the presence of pollution and present several processes analogous to those of vertebrates and mammals [103,104].

**Figure 3:**
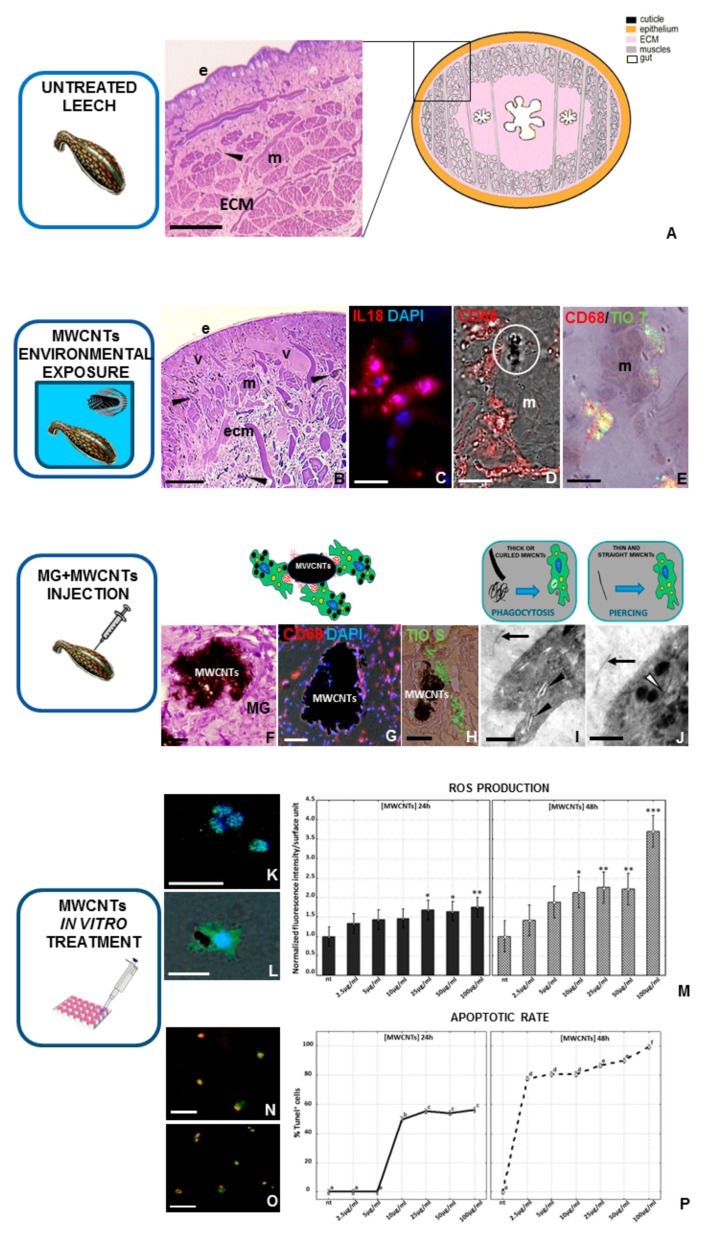
Effects of MWCNT treatment on leech tissues and cultured macrophages. (**A**) Schematic representation and image taken via optical microscope of the cross-sectioned body wall of an untreated leech. Underneath the cuticle and epithelium (e), only a few resident immunocompetent cells (arrow), dispersed in a loose connective (ECM) and predominantly avascular tissue, were detectable around the muscle fibers (m). After MWCNT environmental exposure (**B**–**E**), several new vessels (v) and numerous migrating cells are localized under the epithelium (e) and among muscle fibers (m). Indeed, numerous IL-18^+^ (red in (**C**)) and CD68^+^ macrophages (red in (**D**)) were recruited in the stimulated area, near which MWCNT aggregates were visible (white circle in (**D**)). Moreover, double-staining analyses, using the thioflavin-T method, indicated that CD68^+^ cells were involved in amyloid fibrils production (yellow in (**E**)). In Vivo matrigel (MG) assay (**F**–**J**). In the MG pellet supplemented with MWCNTs, several CD68^+^ (red in (**G**)) and thioflavin-S^+^ (green in (**H**)) macrophages infiltrated the biomatrix, surrounded the nanoparticles aggregates, and produced amyloid fibrils, as represented in the drawing. TEM details show the presence of MWCNTs freely dispersed in the MG (black arrows in (**I**) and (**J**)) or localized in intracellular vesicles (black arrowheads in (**I**)) or cellular cytoplasm (white arrowhead in (**J**)). As shown in the drawings, nanoparticles can be engulfed or pierced inside cells based on their size and their spatial organization. In Vitro MWCNTs treatment (**K**–**P**). Both after 24 and after 48 h, immunofluorescent analyses (**K**,**L**) revealed that macrophage ROS production increases after MWCNTs treatment in a dose-dependent manner, as indicated in the histograms related to the H_2_DCFH-DA fluorescence intensity (**M**). Similarly, TUNEL assays (**N**,**P**) show an increased apoptotic rate after MWCNTs in vitro treatment, as also demonstrated in the graphs, illustrating TUNEL^+^ nuclei percentage. Nuclei in blue (**C**,**G**,**K**,**L**) are counterstained with DAPI. Bar in (**A**) 100 µm; bars in (**B**,**F**,**G**) 50 µm; bar in (**H**): 25 µm; bars in (**N**,**O**) 20 µm; bars in (**C**–**E**,**K**,**L**) 10 µm; bar in (**I**) 2 µm; bar in (**J**) 1 µm. (this figure was reproduced from Girardello et al. [101] under the terms of the Creative Commons Attribution License). * *p* < 0.05, ** *p* < 0.01, *** *p* < 0.001.

In a short period of time (6, 24 h), the potential toxicity of MWCNTs can be directly evaluated on the leech’s body wall, which is predominantly avascular. The cellular arm of leech immunity presents a few immunocompetent cells of myeloid origin, such as macrophages, granulocytes, and NK [105] dispersed in the extracellular matrix surrounding the muscle cells (Figure 3A). 

Moreover, the immune response interestingly involves biological mechanisms, molecules, and cellular pathways highly similar to those already observed in vertebrates [105,106,107,108,109]. All these elements perform fundamental roles in the regulation of cell activation and differentiation, and the consequent responses, triggered by MWCNT treatments, with results being clearly detectable in this invertebrate model.

In fact, the MWCNTs dispersed in water induce noxious effects even at low concentrations and after short periods of exposure. Indeed, optical and ultrastructural analyses of leech tissues revealed that NPs cause a strong inflammatory response, angiogenesis, fibroplasia, immune cell activation, and widespread pro-inflammatory cytokine expression, suggesting that these nanomaterials penetrate the external barriers and mainly enter through the skin. Moreover, MWCNTs not only were observed aggregated and dispersed in the muscular layer, but also in the macrophage-like cytoplasm [101] and after their body entry, the extracellular matrix was widely remodeled. As already observed in leeches as a consequence of the induced inflammation, collagen fibers are reorganized in order to generate a new precise scaffold, which is crucial for driving the development of new vessels and the migration of immunocompetent myeloid cells [109,110]. In particular, after environmental MWCNT exposure (Figure 3B–E), numerous CD68^+^ (Figure 3D) and *Hm*AIF-1^+^ cells express the pro-inflammatory interleukin 18 (IL-18) (Figure 3C), which is involved in angiogenesis and CD45^+^ and CD68^+^ cell migration up to the challenged area. Of note, as observed in vertebrates, these immune events are independent from confounding CNT synthesis by-products such as trace metals [111,112]. Indeed, both atomic absorption spectroscopy and X-ray spectroscopy analyses demonstrate that no metals, such as aluminum oxide, cobalt, and iron, are detectable in the leech’s body wall exposed to MWCNTs, confirming that the observed responses were caused by MWCNTs and not by metal oxide impurities in the exposure solution [101]. The toxic effects of NPs are also detectable by examining the behavior of CD68^+^ macrophages. Indeed, the injection of the matrigel biopolymer (MG) added with MWCNTs into the leech’s body wall (Figure 3F–J), a technique used for specifically isolating and analyzing leech cell populations [113,114,115], revealed that numerous activated macrophages migrate into the MG sponges, attracted to the aggregated nanotubes (Figure 3F). Ultrastructural TEM analyses also showed that MWCNTs are phagocytosed (Figure 3I) or internalized by endocytosis (Figure 3J) and then spontaneously disseminated in the cytoplasm. It has been reported that MWCNTs organized in rigid structures seem to penetrate cellular lipid layers, supporting the in vitro observations conducted in vertebrates [116,117,118]. These mechanisms of diffusion induce toxicity, as demonstrated by the release of ROS. Indeed, many studies revealed that during oxidative stress, the related damages promote protein misfolding that leads to amyloid fibril formation [119,120,121].

Thus, MWCNTs indirectly influence protein aggregation processes, which are often related to several neurodegenerative diseases, in which amyloid fibrils are widely produced. This result is clearly visible in leech tissues, in which, as demonstrated by the thioflavin-T and S colorimetric methods (Figure 3E,H), the amyloid deposition occurs after the MWCNTs/macrophages associations [116]. Interestingly, the IL-18 expression (Figure 3C), concurrently with the thioflavin signal present in CD68^+^ macrophages (Figure 3E), progressively increases in a time-dependent manner after MWCNT treatment [122,123], revealing parallel expression of this cytokine and amyloid materials after macrophage activation. The significant increase in thioflavin-S staining was observed in MWCNT-exposed macrophages in a dose and time-dependent manner and the amyloid fibrils formed a scaffold around the aggregates, confirming that their deposition contains non-self-materials by the formation of a solid barrier. Moreover, the in vitro methods (Figure 3K–P) also validated a correlation between MWCNTs and apoptosis. The increase of ROS production (Figure 3M) is related to apoptotic events, becoming a fundamental indicator for nanomaterial toxicity [124], because at high concentrations ROS are the direct consequence of cell death. In particular, although at lower concentrations of MWCNTs (from 10 to 50 μg/mL) the ROS production is not significantly detectable and not all cells are under oxidative stress, after 100 μg/mL treatment, the ROS release (Figure 3K,L) intensely rises, as does the number of apoptotic cells (Figure 3N–P). The cells treated with low doses and for a short period of time show more tolerance, possibly due to specific scavenger systems with antioxidant abilities capable of controlling the redox state in normal cells [102,125].

The toxicological evidence obtained in the medicinal leech therefore confirms that the dispersion of graphene nanotubes poses potential risks for public health and aquatic environments. The importance of confirming these analyses is linked to the reliability and reproducibility of the data. In this regard, the leech model respects these parameters through implementing the monitoring of the diffusion of MWCNTs in the aquatic environment and their effects as potential stressors on living organisms.

## 5. Conserved Stress and Immune Responses of Annelid Immunocytes to Nanomaterials

For a better comparison, hereby we provide a short synopsis (Table 2) on the major characteristics of our NP-related studies and research outcomes on the cellular immune elements of annelids.

## 6. Conclusions

In this review we have given an account of the recent knowledge about the toxicity of silver and carbon nanomaterials and how they can evoke stress and immune responses in terrestrial and freshwater annelids. Earthworms and their cousin, the leech, are important ecological sentinels, and as such, earthworms are widely used in (eco)toxicological assays, while leeches are less frequently applied. AgNPs and MWCNTs have similarly detrimental effects on those organisms at different biological levels. A similar inflammatory stress response pattern has been observed upon NM exposure. Nothing is known about the protein-corona formation and NP uptake mechanisms in leeches; however, similar PRRs are present in both species [46,103] that might be involved in the NP engulfment. This direction needs further future investigations.

## Figures and Tables

**Table 1:** Summary of toxicological studies of AgNPs in different earthworm species.

Species	AgNPs			Type of Exposure	Exposure Duration	Endpoints	References
	Capping Agent	Size (nm)	Nominal Concentration				
*E. fetida*	PVP and oleic acid	30–50	10, 100, 1000 mg/kg soil	in vivo	28 d	survival, growth, mortality reproduction	[11]
*E. fetida*	PVP	10, 30–50	range of concentrations up to 100 mg/kg soil	in vivo	48 h	avoidance behavior	[16]
	oleic acid	30–50					
	citrate	15–25					
*A. chlorotica*	uncoated	80	various, up to 100 mg/kg soil	in vivo	14 d	uptake, survival, avoidance response, biomass change	[31]
*E. albidus*	PVP	30–50	various, up to 1000 mg/kg for the survival and 600 mg/kg for the gene expression	in vivo	2 d and 6 weeks	survival, reproduction gene expression	[32]
*L. terrestris*	*ND*	20.2 ± 2.5 colloidal: 8.8	various, up to 100 mg/L in water and 100 mg/kg in soil	in vivo	24 h (water) 2, 4, 8 weeks (soil)	mortality, apoptosis	[23]
*E. crypticus*	uncoated	15	different concentrations up to 225 mg/kg	in vivo	0, 3, 7 d	genotoxicity	[40]
*L. rubellus*	uncoated	50	100 and 500 mg/kg in soil	in vivo	10 exposure times, up to 168 h	uptake and elimination	[30]
*E. fetida*	PVP	30–50	500 mg/kg	in vivo	1–14 d	time-course profile of stress and immune related genes enzyme activities	[41]
*E. fetida*	uncoated	10	0–1500 mg/kg	in vivo	4 and 28 d	oxidative stress biomarkers	[42]
*E. fetida*	PVP	83 ± 22	0–5.91 µg/mL	in vitro	24 h	cell viability, cytotoxicity, oxidative, and immune related genes expression	[12]
*E. fetida*	uncoated	15	1–20 µg/mL	in vitro	2, 4, 8, and 24 h	profile of immune and oxidative stress-related genes, protein secretion	[41]
*E. fetida* *E. andrei*	PVP	10	1.25–40 µg/mL	in vitro	24 h	cytotoxicity, apoptosis, genotoxicity oxidative, and immune related gene expression protein secretion	[27]

**Table 2:** Summary of the NP-related factors and effects on annelid sentinels.

Animals	Earthworms (*E. fetida*, *E. andrei*)	Leeches (*Hirudo medicinalis*, *H. verbana*)
Type of NPs	AgNPs [11,12,17,27,44,45]	MWCNTs (9.5 nm external diameter, 1.5 μm length, surface area 250–300 m^2^/g) [101,102,125]
Dose	Please see Table 1.	400 mg/L (in vivo) [101] 2.5, 5, 10, 25, 50, 100 µg/ml (in vitro) [102,125]
Exposure routes	Soil [11,16,30,31,40,41,42]	Surface water [101,102,125]
NP contact	Gut and skin epithelial cells, coelomocytes, mucus [23,27]	Skin epithelial cells, macrophages [101,102,125]
Confounding factors	AgNP oxidation and sulphidiation [7,11,12,13,14,15]	CNT aggregation, metal trace elements [101]
Protein corona	Rich in lysenins [27,45,46]	Not known
Oxidative stress	ROS production, apoptosis [12,23,27]	ROS production, apoptosis [125]
Inflammatory response	Biased immune-related and stress gene (*TLR*, *MyD88*, *MT*, *SOD*, *lysenin*) expression [12,27,41,46]	Immune-related biomarker (CD68, CD45, IL-18, *Hm*AIF-1) [101,102,125]
Cellular clearance of NPs	AgNPs accumulation in amoebocytes [12,66]	MWCNTs accumulation in phagocytic macrophages [101,102,125]
